# Velocity encoded cardiovascular magnetic resonance to assess left atrial appendage emptying

**DOI:** 10.1186/1532-429X-14-39

**Published:** 2012-06-21

**Authors:** Kai Muellerleile, Arian Sultan, Michael Groth, Daniel Steven, Boris Hoffmann, Gerhard Adam, Gunnar K Lund, Thomas Rostock, Stephan Willems

**Affiliations:** 1 Center for Cardiology and Cardiovascular Surgery, University Medical Center Hamburg-Eppendorf, Martinistrasse 52, D - 20246, Hamburg, Germany; 2 Department of Diagnostic and Interventional Radiology, University Medical Center Hamburg-Eppendorf, Martinistrasse 52, D - 20246, Hamburg, Germany

**Keywords:** Left atrial appendage, Atrial fibrillation, Stroke, Cardiovascular magnetic resonance, Transesophageal echocardiography.

## Abstract

**Background:**

The presence of impaired left atrial appendage (LAA) function identifies patients who are prone to thrombus formation in the LAA and therefore being at high risk for subsequent cardioembolic stroke. LAA function is typically assessed by measurements of LAA emptying velocities using transesophageal echocardiography (TEE) in clinical routine. This study aimed at evaluating the feasibility of assessing LAA emptying by velocity encoded (VENC) cardiovascular magnetic resonance (CMR).

**Methods:**

This study included 30 patients with sinus rhythm (n = 18) or atrial fibrillation (n = 12). VENC-CMR velocity measurements were performed perpendicular to the orifice of the LAA. Peak velocities were measured of passive diastolic LAA emptying (e-wave) in all patients. Peak velocities of active, late-diastolic LAA emptying (a-wave) were assessed in patients with sinus rhythm. Correlation and agreement was analyzed between VENC-CMR and TEE measurements of e- and a-wave peak velocities.

**Results:**

A significant correlation and good agreement was found between VENC-CMR and TEE measurements of maximal e-wave velocities (r = 0.61, P < 0.001; mean difference 0 ± 10 cm/s). The a-wave was detectable by VENC-CMR in all patients with sinus rhythm. Correlation was also significant for measurements of peak a-wave velocities between VENC-CMR and TEE (r = 0.71, P < 0.001). There was no significant correlation of LAA emptying velocities with clinical characteristics and only a modest negative correlation of passive LAA emptying with LA function.

**Conclusions:**

The assessment of active and passive LAA emptying by VENC-CMR is feasible. Further evaluation is required of potential future clinical applications such as risk stratification for cardioembolic stroke.

## Background

The left atrial appendage (LAA) is the major source of thrombotic material in patients with cardioembolic stroke [1]. Thrombus formation is promoted by an impaired LAA function with reduced emptying of the LAA [2]. An impaired LAA function is typically related to atrial fibrillation (AF). However, LAA function can also be reduced in patients with sinus rhythm (SR) due to transient stunning after cardioversion or sustained dysfunction after catheter ablation of AF [3,4]. The assessment of LAA emptying aims at estimating the risk for thromboembolic events in several clinical settings and is typically performed by Doppler transesophageal echocardiography (TEE) [1,5]. However, TEE is invasive, requires sedation and is associated with the potential risk of complications in the upper gastro-intestinal-tract [6]. Therefore growing interest has been recently paid to non-invasive techniques for the assessment of LAA emptying [7].

Cardiovascular magnetic resonance (CMR) offers unique features in patients at risk for stroke: Apart from the ability to detect LAA thrombi and to measure LAA dimensions, LA fibrosis quantified by Late Gadolinium Enhancement (LGE) CMR was recently proposed as a novel marker for high-risk patients [8-11]. Furthermore, plaques with retrograde flow in the descending aorta can be assessed by CMR as a potential non-cardiac source for cerebral embolism [12,13]. Velocity-encoded (VENC) CMR serves as the reference method for the quantification of intracardiac flow, indicating a potential use for measurements of LAA flow as a component of a comprehensive CMR protocol in patients at risk for stroke [14]. However, the assessment of LAA function by CMR has not been studied yet. We hypothesized that VENC-CMR could be used to assess LAA emptying velocities. Thus, this study compared measurements of LAA emptying velocities between VENC-CMR and TEE.

## Methods

### Patients

The study was approved by the local ethics committee and all patients gave their written informed consent. TEE and CMR were performed in 30 consecutive patients before scheduled catheter AF ablation for the assessment of LA and pulmonary venous anatomy. TEE was performed as a part of clinical routine to exclude LAA thrombi before AF ablation. CMR and TEE were carried out on the same day. Eighteen (60%) patients with a history of paroxysmal AF were in SR and 12 (40%) patients with persistent AF were in AF when CMR and TEE were performed. Patients were excluded if contraindications for CMR such as implanted pacemakers or severe claustrophobia were present.

### LAA emptying velocities by VENC-CMR

**Figure 1:**
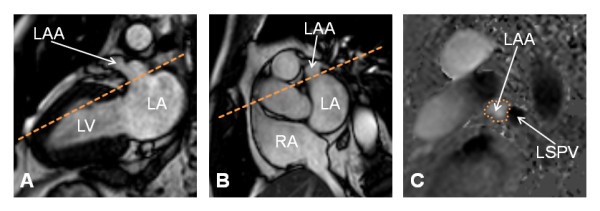
**VENC-CMR slice positioning for LAA flow measurements.** A VENC-CMR slice was positioned (dashed line) perpendicular to the LAA orifice on long-axis (Figure 1A) and short-axis (Figure 1B) cine-CMR views. The region of interest for flow measurements was drawn around the LAA orifice as outlined by the dotted line on the phase VENC-CMR image (Figure 1C). *Abbreviations:* LAA = left atrial appendage, LA = left atrium, LV = left ventricle, RA = right atrium, LSPV = left superior pulmonary vein.

CMR was performed using a 1.5 Tesla system (Achieva, Philips Medical Systems, Best, The Netherlands). All sequences were ECG-triggered and breath-held. Scout images were performed in axial, coronal and sagittal orientation. Standard retrospectively vector-ECG gated cine-CMR was performed in short- and long-axis to assess cardiac volumes and function. Additionally, cine-CMR of the LAA was performed in a long-axis view (Figure 1A) and in the short axis orientation (Figure 1B). Typical imaging parameters of cine-CMR were as follows: pixel spacing 1.56 × 1.56 mm^2^, slice thickness 8 mm, time to repetition = 2.8 ms, echo time = 1.4 ms, flip angle = 60°. A breath-held, retrospectively vector-ECG gated, two-dimensional flow-sensitive phase-contrast gradient-echo sequence was used for VENC-CMR flow measurements perpendicular to the orifice of the LAA (Figure 1). Furthermore, VENC-CMR transmitral flow measurements were performed to facilitate the interpretation of LAA flow curves. Typical imaging parameters of VENC-CMR were as follows: Pixel spacing 1.37 × 1.37 mm^2^, slice thickness 8 mm, time to repetition = 4.5 ms, echo time = 2.9 ms, flip angle = 15°, 50 frames per RR-interval resulted in an effective temporal resolution of 22 ± 8 ms (60% phase percentage), parallel imaging (SENSE reconstruction) with an acceleration factor of 2, encoding velocity was set to 100 cm/s, typical breathhold durations were about 25 seconds.

**Figure 2:**
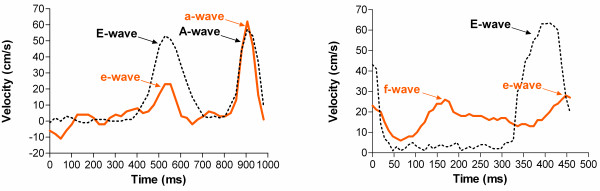
**VENC-CMR measurements of LAA emptying velocities.** LAA flow velocity profiles by VENC-CMR in a patient with SR (Figure 2A) and in a patient with AF (Figure 2B). Transmitral flow velocity profiles (black dotted lines) were superimposed to enable a clear identification of e- and a-waves, respectively. The e-wave was defined by the coincidence with early, passive LA emptying (E-wave, Figure 2A). The a-wave was defined by the coincidence with late, active LA emptying (A-wave, Figure 2A) in patients with SR. Peak e-wave velocity was 23 cm/s and peak a-wave velocity was 62 cm/s in patient A. In patients with AF, a temporal shift between transmitral E-wave and LAA e-wave was observed, that could possibly be related to RR-interval variability or irregular electrical LAA activation. The LAA e-waves were therefore differentiated from f-waves by proximity rather than coincidence to passive diastolic LA emptying (E-wave) in patients with AF (Figure 2B). Peak e-wave velocity was 28 cm/s in patient B. A prominent systolic f-wave was detected in this patient, related to electric activation of the LAA in AF.

VENC- CMR data were analyzed using commercial software (ViewForum workstation R5.1, Philips Medical Systems, Best, The Netherlands). Peak velocities were measured of passive diastolic LAA emptying (e-wave) in all patients. Peak velocities of active, late-diastolic LAA emptying (a-wave) were assessed in patients with sinus rhythm. Figure 2 demonstrates measurements of LAA emptying velocities by VENC- CMR in patients with SR and in patients with AF. All data analyses were repeated with an interval of at least one week to assess intra-observer agreement. An additional reading was performed in all patients by a second observer to assess inter-observer agreement.

### LAA emptying velocities by TEE

**Figure 3:**
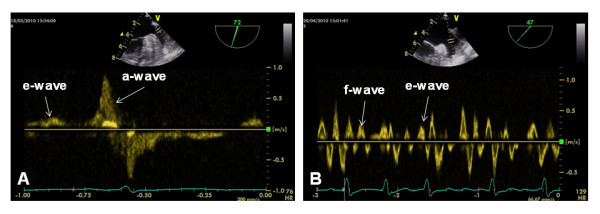
**TEE measurements of LAA emptying velocities.** LAA flow velocity profiles by TEE in a patient with SR (Figure 3A) and in a patient with AF (Figure 3B). Peak e-wave velocity was 28 cm/s and peak a-wave velocity was 73 cm/s in patient A. Mean peak e-wave velocity of five consecutive beats was 26 cm/s in patient B. Prominent f-waves were detected similar to the VENC-CMR flow curve (Figure 3B), corresponding to high-amplitude atrial fibrillation waves in ECG in this patient.

TEE was performed using a Vivid S6 system equipped with a multiplane 5.0 MHz transducer (GE Vingmed Ultrasound, Horten, Norway) as a part of clinical routine to exclude LAA thrombi before AF ablation. All patients received a mild sedation with propofol and additional topical anesthesia using a lidocaine gel in agreement with current recommendations [15]. LAA views were obtained in transverse and longitudinal orientations. LAA flow was assessed using pulsed-wave Doppler by placing the sample volume within the orifice of the LAA (Figure 3). Measurements of five consecutive beats were averaged to define e-wave velocities in patients with AF [16].

### Statistical analysis

Statistical analysis was performed using GraphPad Prism version 5.00 for Windows (GraphPad Software, San Diego, CA, USA). Continuous data are presented as median and interquartile range (IQR). Normality testing was performed using the D'Agostino-Pearson omnibus method. Bland-Altman analysis was used to assess agreement between observers and methods. Correlation between VENC- CMR and TEE was assessed using Pearson`s correlation coefficient. Statistical significance was set to P < 0.05.

## Results

### General

The major patient characteristics are provided in Table 1, indicating a typical AF study population with enlarged LA volumes and preserved LV function (Table 1) [17]. No complications were observed during the CMR and the TEE procedures, respectively. None of the patients had evidence of LAA thrombus on CMR or TEE. All subsequent AF ablation procedures were performed without cerebral complications. VENC-CMR provided sufficient image quality to enable quantification of peak LAA e- and a-wave velocities in all patients. Analysis of VENC-CMR data was fast, requiring less than 5 minutes for complete post-processing and measurements. Median peak e-wave velocity by VENC-CMR was 22 (IQR 16–31) cm/s in the whole study population, 28 (IQR 20–42) cm/s in patients with AF and 18 (IQR 14–24) cm/s in patients with SR, respectively. Median peak a-wave velocity by VENC-CMR was 47 (IQR 33–64) cm/s in patients with SR.

**Table 1:** Patient characteristics

	
**Age (years)**	66 (54–72)
**Female sex**	10 (33)
**BMI**	28 (24–30)
**SR**	18 (60)
**AF**	12 (40)
**Ventricular heart rate (bpm)**	69 (58–78)
**Systolic blood pressure (mmHg)**	140 (128–150)
**Diastolic blood pressure (mmHg)**	80 (75–88)
**LAVi (ml/m**^**2**^**)**	58 (46–73)
**LAEF (%)**	51 (26–64)
**LVEDVi (ml/m**^**2**^**)**	75 (62–81)
**LVESVi (ml/m**^**2**^**)**	31 (27–41)
**LVEF (%)**	55 (47–78)

### Reproducibility of VENC-CMR data analysis

Intra- and inter-observer agreements were strong for data analysis of VENC-CMR peak e-wave velocities with a mean bias of 1 ± 4 and 2 ± 4 cm/s, respectively. There were also strong intra- and inter-observer agreements for data analysis of VENC-CMR peak a-wave velocities with a mean bias of 0 ± 3 and 0 ± 3 cm/s, respectively.

### VENC-CMR vs. TEE

**Figure 4:**
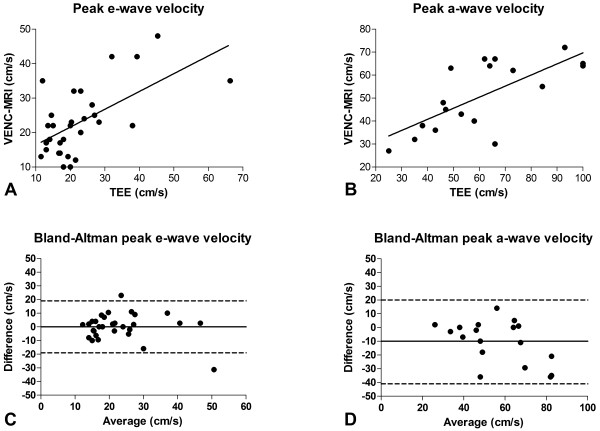
**VENC-CMR vs. TEE.** Figure 4A and C: Correlation and agreement between VENC-CMR and TEE measurements of peak e-wave velocities (r = 0.61, P < 0.001, mean difference 0 ± 10 cm/s). Figure 4B: Correlation for measurements of peak a-wave velocities between VENC-CMR and TEE (r = 0.71, P < 0.001). Figure 4D: Mean underestimation of 10 ± 15 cm/s for peak a-wave velocities (P < 0.05) by VENC-CMR compared to TEE. *On Bland-Altman plots, middle continuous line indicates mean bias, and dashed lines above and below indicate 95% confidence intervals.*

A significant correlation was found between VENC-CMR and TEE measurements of peak e-wave velocities (r = 0.61, P < 0.001) with a mean difference 0 ± 10 cm/s (Figure 4A and C) in the whole study population. No significant correlation was found between VENC-CMR and TEE in a separate analysis of patients with SR (r = 0.33, P = 0.17) and AF (r = 0.58, P = 0.06), respectively. VENC-CMR permitted identification of the a-wave in all patients with sinus rhythm. Correlation was also significant for measurements of peak a-wave velocities between VENC-CMR and TEE (r = 0.71, P < 0.001, Figure 4B). However, a mean difference of 10 ± 15 cm/s (P < 0.05) was found between VENC-CMR and TEE peak a-wave velocities, indicating a potential systematic underestimation of peak a-wave velocities by VENC-CMR (Figure 4D).

### LAA emptying vs. Clinical and CMR parameters

There was no significant correlation of VENC-CMR peak e- and a-wave velocities with heart rate (r = 0.31 and r = 0.18), body-mass-index (r = −0.01 and r = 0.26), systolic (r = −0.01 and r = 0.06) and diastolic (r = 0.25 and r = 0.21) blood pressure, respectively (P = ns). Furthermore, there was no significant correlation of VENC-CMR peak e- and a-wave velocities with LV end-diastolic (r = 0.26 and r = 0.03), LV end-systolic (r = 0.25 and r = −0.10) and LA (r = 0.20 and r = −0.21) volume indices or LV ejection fraction (r = −0.14 and r = 0.26). There was no significant correlation between peak LAA e-wave and mitral valve E-wave velocities (r = 0.31, P = 0.15) as well as between peak LAA a-wave and mitral valve A-wave velocities (r = 0.23, P = 0.44). A modest negative correlation was found between peak LAA e-wave velocities and LA ejection fraction (r = −0.46, P < 0.05) but not between peak LAA a-wave velocities and LA ejection fraction (r = −0.01, P = 0.99).

## Discussion

This study compared measurements of LAA emptying velocities between VENC-CMR and TEE in thirty consecutive patients. The major findings were:

· Inter- and intra-observer agreement was strong for data analysis of VENC-CMR LAA emptying velocity measurements.

· There was a significant correlation for measurements of peak LAA e- and a-wave velocities between VENC-CMR and TEE.

· There was no significant correlation of LAA emptying velocities with clinical characteristics and only a modest negative correlation of passive LAA emptying with LA function.

### Passive LAA emptying

Passive LAA emptying is primarily related to a suction effect of passive LA emptying in early diastole in SR as well as in AF [13,14]. In SR, the e-wave velocity is a surrogate parameter for passive LAA emptying and is typically lower than active emptying velocities (Figure 3A) [14]. Being of little relevance in SR under physiological conditions, passive LAA emptying gains importance in situations with impaired active LAA emptying such as atrial stunning [3,4,13,14]. In patients with AF, LAA emptying is inherently passive. Doppler TEE differentiates variable systolic f-waves related to electrical LAA activation from diastolic e-waves related to passive LA emptying, similar to e-waves in SR [16,18]. Principle methodological differences between VENC-CMR and TEE have to be considered for interpreting LAA flow curves in AF: CMR data acquisition is distributed to several cardiac cycles in segmented ECG-gated sequences. Thus, LAA flow curves by VENC-CMR represent composed data from several cardiac cycles with variable RR-intervals and degrees of electrical LAA activation. We defined VENC-CMR e-waves in AF by proximity with superimposed passive LA emptying to facilitate a clear differentiation from systolic f-waves (Figures 2 and 3). Nevertheless, it is important to highlight the inherent difference between distinct e- and f-waves in TEE compared to the averaged velocity peaks in VENC-CMR curves despite a similar appearance.

Despite the technically related differences, our findings indicate that VENC-CMR measurements of passive LAA are feasible. However, the non-significant correlation between CMR and TEE measurements within the subgroups of patient with SR and AF requires further evaluation in larger populations. Nevertheless, recent data indicate a potential role for CMR measurements of LAA dimensions and function in patients at risk for embolic stroke. Beinart et al. reported an independent predictive value of LAA dimensions for embolic cerebral events in a study with 144 patients [10]. Considering the well established association between reduced LAA emptying velocities and thrombus formation in the LAA, VENC-CMR could be an attractive adjunctive non-invasive tool in this context [1,2]. A comprehensive CMR protocol including measurements of LA fibrosis, LAA dimensions and passive LAA emptying velocities could potentially improve risk stratification in patients with AF [8-11]. Furthermore, Donal et al. reported a high specificity of e-wave velocities by TEE during AF to predict recovery of active LAA emptying after AF ablation [19]. Thus, VENC-CMR measurements of passive LAA velocities could also be used for the non-invasive prediction of potential recovery of LAA contractility after successful AF ablation.

### Active LAA emptying

Active LAA emptying is related to LAA contraction following the electric activation of the LA and LAA in SR. The LAA a-wave occurs simultaneously with the A-wave of transmitral flow immediately after the surface ECG P-wave. LAA a-wave velocity is a surrogate parameter for active LAA emptying [16,18]. A reduced a-wave velocity can be present in SR as transient stunning after cardioversion or AF ablation, but is also related to ageing [3,13,16,20]. However, since TEE is an invasive procedure, data are limited on time course, potential predictors and modulators of LAA stunning after catheter ablation of AF [16,18]. Of particular interest are patients after ablation of persistent AF. In these patients, extended ablation strategies are necessary, which have the potential risk of an inadvertent electric isolation of the LAA [4,21]. Thus, there is a need for a non-invasive diagnostic tool permitting the proof of active LAA emptying after persistent AF ablation.

We found a significant correlation of a-wave velocities between VENC-CMR and TEE, indicating the feasibility of LAA emptying measurements using VENC-CMR. However, there was a mean underestimation of 10 ± 15 cm/s for peak a-wave velocities by VENC-CMR compared to TEE. This finding is in agreement with recent data on underestimation of vascular and valvular peak velocities by VENC-CMR compared to ultrasound measurements, upcoming advanced VENC sequences may overcome this discrepancy in future [22]. Nevertheless, VENC-CMR measurements appear to be potentially useful to differentiate low-risk patients with fully recovered active LAA emptying from high-risk patients with permanently missing active LAA emptying. Furthermore, VENC-CMR could be used in combination with LGE CMR to longitudinally monitor recovery of active LAA emptying and its relationship to the extent of ablation lesions [23,24].

### LAA emptying and clinical characteristics

We did not find a significant correlation between passive LAA emptying velocities and clinical parameters such as heart rate, body-mass-index, blood-pressure, LV volumes and function. Furthermore, LAA emptying velocities were only weakly associated with LA function. These findings are in agreement with recent data from TEE, underscoring that LAA emptying cannot be predicted by clinical characteristics or by assessing LA function [16,18]. Thus, LA and LAA function require separate assessment; VENC-CMR seems to be a promising tool in this context.

In summary, our study demonstrated the feasibility of non-invasive LAA emptying velocity measurements by VENC-CMR. Nevertheless, some principle technical differences between VENC-CMR and TEE have to be considered: Accurate VENC-CMR measurements require blood flow perpendicular to slice orientation, whereas Doppler TEE measurements depend on the correct alignment of probe orientation with blood flow. Thus, differences in sampling locations and slice/probe orientation with respect to LAA blood flow could have influenced the agreement between VENC-CMR and TEE measurements. VENC-CMR has the principle advantage of free slice orientation compared to the limited TTE acoustic window. In contrast, the superior temporal and spatial resolution constitutes a potential advantage of TEE. Furthermore, transthoracic echocardiography (TTE) was recently introduced as another non-invasive alternative to TEE for the assessment of LAA emptying [7].

However, the true incremental value of VENC-CMR compared to other techniques is the combinability of LAA emptying measurements with novel CMR features such as the assessment of LA fibrosis [11]. VENC-CMR emptying measurements require one extra breathhold, adding less than one minute to a standard CMR protocol. Thus, there are several potential future applications of VENC-CMR LAA emptying measurements:

First, VENC-CMR LAA flow measurements could be integrated into a comprehensive CMR protocol for the evaluation of patients at risk for stroke. Such a protocol could incorporate information on LA dimensions and function, LAA emptying, LA fibrosis as well as on aortic plaque morphology and flow characteristics to improve current risk stratification for stroke [8-13]. Second, VENC-CMR LAA flow measurements could be combined with MR angiographies of the LA and pulmonary veins that are frequently performed in clinical routine before AF ablation procedures [25]. In this context, LAA flow measurements could be helpful to predict recovery of LAA function after successful AF ablation. Third, VENC-CMR LAA flow measurements could be used in combination with LGE CMR to assess LAA dysfunction and its association with LA ablation lesions after AF ablation. Furthermore, VENC-CMR LAA flow measurements could be helpful in patients who are candidates for percutaneous LAA closure procedures to simultaneously assess LAA morphology and function [26]. However, further evaluation of VENC-CMR LAA flow measurements is required in larger populations before clinical implementation.

### Limitations

This feasibility study is mainly limited by the small study population. Furthermore, recent findings from transmitral flow measurements indicate an improved accuracy of intracardiac flow-measurements using three-dimensional VENC-CMR sequences [27]. The use of a conventional two-dimensional VENC-CMR sequence constitutes a potential limitation of this study. In addition, future studies on VENC-CMR LAA flow measurements should address not only reproducibility of data analysis but also reproducibility of data acquisition. Nevertheless, our findings clearly demonstrated the feasibility of LAA emptying measurements by VENC-CMR.

## Conclusions

The assessment of active and passive LAA emptying by VENC-CMR is feasible. Thus, VENC-CMR has the potential to non-invasively identify patients with impaired LAA function who are at risk for stroke.

## Abbreviations

BMI, Body-mass-index; SR, Sinus rhythm; AF, Atrial fibrillation; Bpm, Beats per minute; LAVi, Left atrial volume index; LVEDVi, Left ventricular end-diastolic volume index; LVESVi, Left ventricular end-systolic volume index; LAEF, Left atrial ejection fraction; LVEF, Left ventricular ejection fraction;  , Numbers are median (interquartile range) for continuous and n (% of total column number) for categorical data.

## Competing interests

The authors declare that they have no competing interests.

## Authors’ contributions

All authors participated in editing and drafting of the manuscript. KM, AS, MG, GA, TR and SW contributed to concept, design and coordination of the present study. KM, MG, GKL and GA were involved in CMR data acquisition, analysis and interpretation. KM, AS, DS and BH were involved in TEE data acquisition, analysis and interpretation. All authors read and approved the final manuscript.
